# Genomic Selection for Economically Important Traits in Dual-Purpose Simmental Cattle

**DOI:** 10.3390/ani15131960

**Published:** 2025-07-03

**Authors:** Xiaoxue Zhang, Dan Wang, Menghua Zhang, Lei Xu, Xixia Huang, Yachun Wang

**Affiliations:** 1 College of Animal Science, Xinjiang Agricultural University, Urumqi 830052, China; zhangxiaoxue0726@163.com (X.Z.); wangdan01100330@163.com (D.W.); zhangmenghua810@126.com (M.Z.); q609468041@sina.com (L.X.); 2 Key Laboratory of Animal Genetics, Breeding and Reproduction of Ministry of Agriculture and Rural Affairs, National Engineering Laboratory of Animal Breeding, College of Animal Science and Technology, China Agricultural University, Beijing 100193, China

**Keywords:** single-step GBLUP, milk-production traits, reproduction traits, growth traits, Chinese Simmental cattle

## Abstract

Genomic selection (GS) is a new landmark method in modern animal breeding programs, and it has become a tool for routine genetic evaluation regarding dual-purpose cattle breeding. In this study, we employed data on milk-production, reproduction, and growth measurements of dual-purpose Simmental cows during the period 1987–2022 from two large-scale farms in Northwest China. For this purpose, we used a single-trait model based on the A-array PBLUP and H-array ssGBLUP to perform genetic evaluation of milk-production, reproduction, and growth traits by applying the restricted maximum likelihood (REML) methods. The results revealed that the heritability based on the additive genetic correlation matrix was approximately 0.09–0.31 for milk-production traits, 0.03–0.43 for reproduction traits, and 0.13–0.43 for growth traits. In addition, the heritability based on the genome–pedigree association matrix was similarly 0.09–0.32 for milk-production traits, 0.04–0.44 for reproductive traits, and 0.14–0.43 for growth traits. In the entire population, the reliability of genomic estimated breeding values (GEBVs) increased by 0.6–3.2%, 0.2–2.4%, and 0.5–1.5% for milk-production, reproductive traits, and growth traits, respectively. In the genotyped population, the reliability of GEBV for milk-production and reproduction traits increased by 1.6–4.0% and 0.4–3.6%, respectively, whereas the reliability of GEBV for growth traits decreased by 12.0–17.0%. These results suggest that the construction of an H-matrix with ssGBLUP could improve the heritability and reliability of breeding values for milk-production and reproduction traits. However, the advantage was not evident for growth traits in smaller populations. The present results thus provide a basis for future application of genomic genetic evaluation of dual-purpose Simmental cattle, providing data support for the selection and marketing of excellent breeding bulls, thereby helping to establish a basis for their independently bred breeding bull.

## Simple Summary

Dual-purpose Simmental cattle are receiving increasing attention, and their performance has improved with the advent of combining advanced breeding techniques with production practices. Therefore, new options are needed to increase the accuracy of genetic selection of dual-purpose Simmental cattle. The objective of this study was to estimate the genetic parameters of milk-production, reproduction, and growth traits and compare the accuracy analysis of the estimated breeding value between the traditional best linear unbiased prediction (BLUP) method and genomic selection, using the single-step genomic BLUP (ssGBLUP) method. Through this research, we found that the heritability of milk-production traits, reproduction traits, and growth traits was generally low to moderate; in the analysis of estimated breeding value accuracy, the ssGBLUP method was superior to the pedigree-based best linear unbiased prediction (PBLUP). These findings demonstrate the feasibility of applying the ssGBLUP method in breeding programs for dual-purpose Simmental cattle populations.

## 1. Introduction

In 2023, the Xinjiang region alone had a cattle stock of 8.16 million heads, with milk production of 2.33 million tons and beef production of 0.58 million tons, which have increased by 18.05%, 4.6%, and 18.22%, respectively, from last year (https://www.stats.gov.cn/sj/ndsj/ (accessed on 10 March 2025)). Given the high demand for both dairy and meat products in Northwest China, dual-purpose breeds have received increasing attention due to their ability to produce high-quality milk and meat simultaneously, making them ideal for diversified agricultural operations. In recent years, the objectives of domestic and foreign cattle-breeding practices have witnessed continuous improvement. For instance, in addition to these production [1,2,3,4] and conformation traits [5,6,7,8], functional traits related to reproduction (e.g., calving ease) [3,9] and longevity (e.g., productive life) [10,11,12], have also received increasing emphasis. The continuous optimization of breeding objectives and selection indices, coupled with the increasing adoption of balanced breeding strategies, has promoted cattle-breeding programs toward better economic returns for livestock producers.

**Figure 1:**
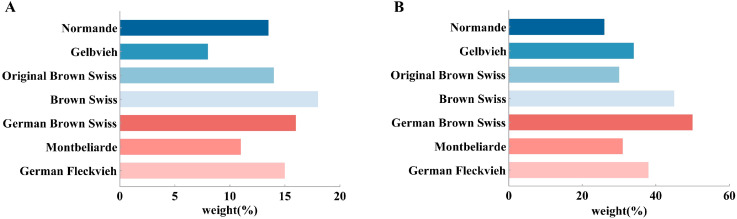
The weighting of milk-production traits (**A**) and reproduction traits (**B**) in the total performance index varies across cattle breeds.

When considering the total performance index (TPI) of cattle breeds in each country, milk-production traits primarily include the yield, fat rate, protein rate, fat, and protein of the milk. The weight of the milk-production traits of the German brown cows was the highest, at 50%, and those of the Normandy cows were the lowest, at 26% (Figure 1A). The reproduction traits mainly included fecundity, calving ease, and the interval between the first mating and the first calf. Of these traits, the weight of the reproduction traits of the Swiss brown cows was the highest, at 18%, and that of the German yellow cows the lowest, at 8% (Figure 1B) [13,14,15,16,17,18]. China’s “2024 Summary of Genetic Evaluation of Chinese Beef and Dairy-meat Dual-purpose Breeding Bulls” introduced the China Beef Selection Index (CBI), wherein newborn weight and weight at 6 months of age constituted 10% and 40% of the score, respectively. TPI for dual-purpose cattle included 40% for milk-production traits, suggesting that both milk-production and reproduction traits are important in breeding dual-purpose cattle [19].

The genetic evaluation of the dual-purpose Simmental cattle populations in the Xinjiang region has long been based mainly on conventional quantitative genetic methods (i.e., the collection of genealogical information and phenotypes). However, errors or incomplete recording of genealogical and phenotypic data in pastures often reduce the accuracy of estimated breeding values, which, in turn, slows down the genetic progress and reduces production efficiency. With the advent of genome-wide high-density marker microarrays, genomic data can be applied to predict individual phenotypes that have been widely used in livestock breeding. Conventional methods utilize data only from genotyped individuals, whereas the ssGBLUP method can integrate pedigree and phenotypic information from non-genotyped animals [20,21], enabling early selection based on GEBVs, thereby reducing the generation interval, accelerating genetic progress, and lowering rearing costs [22]. In the present study, based on the pedigree and genomic information, genetic evaluations were conducted on milk-production, reproduction, and growth traits of dual-purpose Simmental cows in the Xinjiang region of China. This study aims to (1) estimate heritability, genetic correlation, and phenotypic correlation of milk-production, reproduction, and growth traits; and (2) compare the accuracy of EBV and GEBV between the whole population and the genotyped subpopulation. It is hoped that the results of this study will provide data support for the adjustment of the dual-purpose Simmental cows breeding program, as well as lay the foundation for the construction of a comprehensive selection index for these cows in the study region.

## 2. Materials and Methods

### 2.1. Phenotypic Data and Pedigree

All the experimental cattle belonged to the dual-purpose Simmental cattle resource population who were raised at Xinjiang Hutubi farm (data collected during 1987–2022) and the Kekedala Chuangjin farm (data collected during 2019–2022) in Northwest China. The traits of milk-production, reproduction, and growth (for newborn and 6-month-old animals) were collected. Nine milk-production parameters were assessed, categorized as 305-day milk yield (305MY), milk fat percentage (MFP), milk fat yield (MFY), milk protein percentage (MPP), milk protein yield (MPY), lactose percentage (LP), total solids rate (TSR), milk urea nitrogen (MUN), and somatic cell score (SCS). The 12 reproduction traits assessed included the age at first service in heifer (AFS), age at their first pregnancy in heifer (AFP), age at first calving in heifer (AFC), interval from the first service to the conception in heifer (FSTCh), gestation length in heifer (GLh), number of services in heifer (NSh), conception rate for the first service in heifer (CRh), interval from the first service to conception in cow (FSTCc), gestation length in cow (GLc), number of services in cow (NSc), conception rate for the first service in cow (CRc), and calving interval in cow (CIc). The 12 growth traits assessed in the study included body height (BH), body length (BL), chest girth (CG), leg circumference (LC), cannon circumference (CC), and body weight (BW) at two stages (newborn and six-month-old). The milk-production traits and reproduction traits were subjected to descriptive statistical analysis for the phenotypic data, and are presented in Tables S1–S3.

The pedigree file employed for the analysis included data for 30,490 animals, and each animal was traced back four generations. In the full datasets, more than 59 sires had ≥100 offspring, and 1 sire had a maximum of 1464 offspring with records, whereas 124 sires had only 1 offspring. More than 6291 dams had ≥2 offspring.

### 2.2. Genotype Data

Blood samples were collected from the tail vein of dual-purpose Simmental cattle using 10 mL of EDTA anticoagulation blood collection tubes (spotting the blood spot card). A total of 1048 dual-purpose Simmental cattle were genotyped by using the GeneSeek GGP Bovine 100K array (Illumina, San Diego, CA, USA), comprising 94,693 SNPs. Quality control was performed via PLINK 1.9 software [23]. The quality control requirements were as follows: (1) exclusion of individual genotype call rates less than 90%; (2) exclusion of single-SNP genotype call rates less than 90%; (3) exclusion of significant deviations from the Hardy–Weinberg equilibrium (*p* < 10^−6^); and (4) exclusionof minor allele frequencies (MAFs) < 0.05. After quality control, we ultimately utilized 83,489 SNPs for further study.

### 2.3. Statistical Analysis

#### 2.3.1. Significance Test of the Influence Factors

The fixed effects were determined by testing the significance of factors affecting milk-production, reproduction traits and growth traits using the GLM procedure of SAS 8.1 software. The model was as follows:

(1) $$y_{ijklmn}=\mu +{Herd}_{i}+{Parity}_{j}+{Year}_{k}+{Season}_{l}+{Lactation}_{m}+e_{ijklmn,}$$

where y is the response of the milk-production trait; μ is the general mean; ${Herd}_{i}$ is the effect of the herd (*i* = 1, 2); ${Parity}_{j}$ is the parity number (*j* = 1, 2, 3, 4, or ≥5); ${Year}_{k}$ is the effect of the calving year (*k* = 2013–2016, 2017–2019, 2020, 2021, 2022); ${Season}_{l}$ is the effect of the calving season (*l* = Spring—March, April, and May; Summer—June, July, and August; Autumn—September, October, and November; Winter—December, January, and February); ${Lactation}_{m}$ is the effect of the lactation period (*m* = 0–100 days, 101–201 days, and ≥201 days); and $e_{ijklmn}$ is the effect of the random residual.

(2) $$y_{ijklmn}=\mu +{Herd}_{i}+{Parity}_{j}+{BS1}_{k}+{BSn}_{l}+{SY1}_{m}+{SYn}_{n}+{SS1}_{o}+{SSn}_{p}+e_{ijklmnopq,}$$

where y is the observation for the reproduction trait; μ is the general mean; ${Herd}_{i}$ is the effect of the herd (*i* = 1, 2); ${Parity}_{j}$ is the parity number (*j* = 1, 2, 3, 4, or ≥5); ${BS1}_{k}$ and ${BSn}_{l}$ is the effect of the breeder (*k* and l = 1–15); ${SY1}_{m}$ and ${SYn}_{n}$ is the effect of service year (*m* and *n* = 2011–2014, 2015–2016, 2017, 2018, 2019, 2020, 2021, and 2022); ${SS1}_{o}$ and ${SSn}_{p}$ is the effect of service season (*o* and *p* = Spring—March, April, and May; Summer—June, July, and August; Autumn—September, October, and November; Winter—December, January, and February); and $e_{ijklmnopq}$ is the effect of the random residual.

(3) $$y_{ijklmn}=\mu +{Year}_{i}+{Month}_{j}+{Sex}_{k}+e_{ijkl,}$$

where y is the observation for growth trait; ${Year}_{i}$ is the effect of the birth year (*i* = 1987–2000, 2001–2006, 2007–2008, 2009–2010, 2011–2012, 2013–2014, 2015–2019); ${Month}_{j}$ is the effect of the birth month (*j* = 1–12); ${Sex}_{k}$ is the effect of sex (*k* = 2); and $e_{ijkl}$ is the effect of random residual. 

#### 2.3.2. Single-Trait Animal Model

A single-trait animal model, the BLUPF90 package, and the REML method were used to estimate the (co) variance components. Effects showing significant influence on each trait are selected as fixed effects in variance component estimation. The model used was as follows:

(4) $$\text{Milk-production}\;\mathrm{traits}:\;y=Xb+Z_{1}a+Z_{2}pe+e,$$

(5) $$\mathrm{Reproduction}\;\mathrm{traits}:\;y=Xb+Z_{1}a+e;y=Xb+Z_{1}a+Z_{2}pe+e,$$

(6) $$\mathrm{Growth}\;\mathrm{traits}:\;y=Xb+Z_{1}a+e;y=Xb+Z_{1}a+Z_{3}m+e,$$

where y is the vector of observation for milk-production, reproduction, and growth trait; b is the vector of fixed effects; a is the vector of random additive genetic effect; pe is the vector of individual permanent environmental effect; m is the vector of maternal genetic effects; e is the vector of random residual; and X, $Z_{1}$, $Z_{2}$, and $Z_{3}$ are the incidence matrices related to fixed effects, random additive genetic, individual permanent environmental effects, and maternal genetic effects, respectively.

The additive genetic effect model uses two genetic variance–covariance structures: for pedigree-based best linear unbiased prediction (PBLUP), a~N (0,$A\sigma_{a}^{2}$), A and $\sigma_{a}^{2}$ represent the pedigree-based additive genetic relationship matrix and additive genetic variance, respectively; for ssGBLUP, a~N (0,$H\sigma_{a}^{2}$), H is the pedigree–genomic relationship matrix. The H matrix was calculated using the following formula:

(7) $$H=\left[\begin{array}{cc} A_{11}-A_{12}A_{22}^{-1}A_{21}+A_{12}A_{22}^{-1}GA_{21} & A_{12}A_{22}^{-1}G\\ GA_{22}^{-1}A_{21} & G \end{array}\right],$$

where the subscripts 1 and 2 of *A* represent non-genotyped and genotyped animals, respectively. G is the genomic relationship matrix, and the calculation formula is $G=MM^{\prime }/2\sum_{k=1}^{m}p_{k}\left(1-p_{k}\right)$, where M is the relationship matrix of SNP effects, $\left(0-2p_{j}\right)$, $\left(1-2p_{j}\right)$, and $\left(2-2p_{j}\right)$ stand for 11 homozygote, the 12 or 21 heterozygote, and the 22 homozygote, $p_{j}$ is the minor allele frequency of the *j*th SNP; m is the number of markers; and $p_{k}$ is the allele frequency of the *k*th SNP. Therefore, the formula for $H^{-1}$ is as follows:

(8) $$H^{-1}=A^{-1}+\left[\begin{array}{cc} 0 & 0\\ 0 & G^{-1}-A_{22}^{-1} \end{array}\right],$$

Heritability ($h^{2}$) for each trait was calculated using the following formula:

(9) $$h^{2}=\sigma_{a}^{2}/\sigma_{p}^{2},\;{SE}^{2}=\left[\frac{\sigma_{a}^{2}}{\sigma_{p}^{2}}\right]\left[\frac{Var\left(\sigma_{a}^{2}\right)}{{\left(\sigma_{a}^{2}\right)}^{2}}+\frac{Var\left(\sigma_{p}^{2}\right)}{{\left(\sigma_{p}^{2}\right)}^{2}}-\frac{Cov\left(\sigma_{a}^{2},\sigma_{p}^{2}\right)}{\sigma_{a}^{2}\sigma_{p}^{2}}\right]$$

where $\sigma_{a}^{2}$ is additive genetic variance; $\sigma_{p}^{2}$ is total phenotypic variance, $\sigma_{p}^{2}=\sigma_{a}^{2}+\sigma_{e}^{2}$; and SE is the standard error of heritability.

#### 2.3.3. Two-Trait Animal Model

The matrix of the two-trait animal model is as follows:

(10) $$\left[\begin{array}{c} y_{1}\\ y_{2} \end{array}\right]=\left[\begin{array}{cc} X_{1} & 0\\ 0 & X_{2} \end{array}\right]\left[\begin{array}{c} \beta_{1}\\ \beta_{2} \end{array}\right]+\left[\begin{array}{cc} Z_{1} & 0\\ 0 & Z_{2} \end{array}\right]\left[\begin{array}{c} a_{1}\\ a_{2} \end{array}\right]+\left[\begin{array}{c} e_{1}\\ e_{1} \end{array}\right],$$

where $y_{i}$ is the vector of observations for the *i*th trait; $\beta_{i}$ is the vector of fixed effects for the *i*th trait; $a_{i}$ is the vector of random additive genetic effect for the *i*th trait; $e_{i}$ is the vector of random residual for the *i*th trait; $X_{i}$ and $Z_{i}$ are the incidence matrices related to fixed effects and individual random additive genetic, respectively.

Correlation for each trait was calculated using the following formula:

(11) $$\mathrm{Genetic}\;\mathrm{correlation}:\;r_{A}=\frac{Cov\left(a_{i},a_{j}\right)}{\sqrt{\sigma_{a_{i}}^{2}\sigma_{a_{j}}^{2}}};\;\mathrm{Phenotypic}\;\mathrm{correlation}:\;r_{P}=\frac{Cov\left(p_{i},p_{j}\right)}{\sqrt{\sigma_{p_{i}}^{2}\sigma_{p_{j}}^{2}}},$$

where $Cov\left(a_{i},a_{j}\right)$ is the genetic covariance between the *i*th and *j*th traits; $Cov\left(p_{i},p_{j}\right)$ is the phenotypic covariance between the *i*th and *j*th traits; $\sigma_{a_{i}}^{2}$ and $\sigma_{a_{j}}^{2}$ are the genetic variance for the *i*th and *j*th traits; and $\sigma_{p_{i}}^{2}$ and $\sigma_{p_{j}}^{2}$ are the phenotypic variance for the *i*th and *j*th traits.

#### 2.3.4. Reliability of Breeding Value Estimates

The formula for calculating the reliability of breeding value estimates is as follows: $R_{EBV}^{2}=1-{SE}^{2}/Var\left(\sigma_{a}^{2}\right)$.

The reliability calculation formula for GEBV is as follows:

(12) $$R_{GEBV}^{2}=\frac{{Cor}^{2}\left(GEBV,y\right)}{r_{y}^{2}}=\frac{{COV}^{2}\left(GEBV,y\right)}{V_{GEBV}V_{a}}=\frac{{Cor}^{2}\left(GEBV,a+e\right)}{V_{GEBV}V_{y}r_{y}^{2}},$$

(13) $$R_{GEBV}^{2}=\frac{{Cov}^{2}\left(GEBV,a\right)}{V_{GEBV}V_{a}}={Cor}^{2}\left(GEBV,a\right),$$

where $R_{EBV}^{2}$ is the reliability of EBV; $R_{GEBV}^{2}$ is the reliability of GEBV; $\frac{Cor\left(GEBV,y\right)}{r_{y}^{2}}$ is the correlation of the GEBV and phenotypic value; and ${Cov}^{2}\left(GEBV,a\right)$ is the square of the correlation coefficient for the GEBV and phenotypic value.

## 3. Results

### 3.1. Results of Fixed-Effects Analysis of Variance

#### 3.1.1. Significance Testing of Factors Affecting Milk-Production Traits

The significance test results of the variance analysis for milk-production traits are presented in Table 1. The effects of farm and calving year on 305MY, MFP, MPY, MPP, MPY, LR, TSR, MUN, and SCS were statistically significant (*p* < 0.01). Parity showed a significant impact on 305MY and MFY (*p* < 0.05) and statistically significant effects on all other traits (*p* < 0.01). The calving season significantly influenced 305MY and MFY (*p* < 0.05) and had a statistically significant effect on MFP, MPP, MPY, TSR, and MUN (*p* < 0.01). Different lactation periods showed extremely significant effects on MFP, MPP, LR, MUN, and SCS (*p* < 0.01).

#### 3.1.2. Significance Testing of Factors Affecting Reproduction Traits

The significance test results of the variance analysis for reproduction traits are listed in Table 2. The effects of the farm on AFSh, GLh, CIc, and GLc were extremely significant (*p* < 0.01). The first breeding technician had an extremely significant effect on CRh and CRc (*p* < 0.01). The last breeding technician showed extremely significant effects on AFCh, AFPh, FSTCh, NSh, FSTCc, and NSc (*p* < 0.01). The first service year had extremely significant effects on AFSh, CRh, and CRh (*p* < 0.01). The last service year showed extremely significant effects on AFCh, AFPh, FSTCh, GLh, NSh, CIc, FSTCc, GLc, and NSc (*p* < 0.01). The first service season had extremely significant effects on AFSh and CRc (*p* < 0.01). The last service season had extremely significant effects on AFCh, FSTCh, GLh, FSTCc, GLc, and NSc (*p* < 0.01). Different parity displayed extremely significant effects on CIc, FSTCc, GLc, NSc, and CRc (*p* < 0.01).

#### 3.1.3. Significance Testing of Factors Affecting Growth Traits

The significance test results of the variance analysis for growth traits are depicted in Table 3. Regarding the results of body size and weight measurements for newborns, the year of birth and sex showed statistically significant effects on all traits (*p* < 0.01), while the month of birth had extremely significant effects on CG, LC, CC, and BW (*p* < 0.01). Regarding the results of body size and weight at 6 months of age, the year of birth and sex had statistically significant effects on BH, BL, CG, CC, and BW (*p* < 0.01), whereas the month of birth had extremely significant effects on all traits (*p* < 0.01).

### 3.2. Estimation of the Variance Components and Heritability

#### 3.2.1. Milk-Production Traits

**Figure 2:**
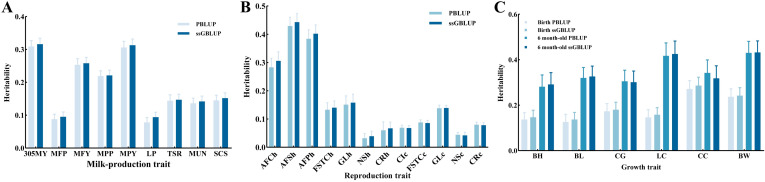
Estimates of heritability for milk-production (**A**), reproduction (**B**), and growth traits (**C**) in different matrices.

The estimated variance components and heritability of the milk-production traits under different matrices are illustrated in Figure 2A and Supplementary Table S4. Generally, the estimated heritability of milk-production traits ranged from 0.078 to 0.316, which belonged to low-to-medium heritability. The heritability estimated by ssGBLUP was higher than that estimated by PBLUP. In different matrices, the traits with the largest heritability were 305MY (0.316) and MPY (0.313), estimated by ssGBLUP, whereas the smallest trait was LP (0.078), estimated by PBLUP. The standard errors of the heritability estimates of different matrices were similar and low (0.015–0.019), indicating consistent accuracy of the heritability estimates.

#### 3.2.2. Reproduction Traits

The estimated variance components and heritability of the reproduction traits under different matrices are illustrated in Figure 2B and Supplementary Table S5. Generally, the estimated heritability of reproduction traits ranged from 0.032 to 0.158, which belonged to low heritability (excluding AFCh, AFSh, and AFPh). The heritability estimated by ssGBLUP was higher than that estimated by PBLUP (excluding CIc, FSTCc, and GLc). In different matrices, the trait with the largest heritability was AFSh (0.443), estimated by ssGBLUP, whereas the smallest trait was NSh (0.032), estimated by PBLUP. The standard errors of the heritability estimates of different matrices were similar and low (0.009–0.032), as indicated by the consistency of the heritability estimates.

#### 3.2.3. Growth Traits

Variance components and heritability estimates for the growth traits under different matrices are shown in Figure 2C and Supplementary Table S6. Overall, the heritability estimates for the growth traits ranged from 0.137 to 0.432, which indicates moderate-to-high heritability. For the newborn growth traits, CC (0.286) and BL (0.137) had the largest and smallest heritability estimates, respectively, under the different matrices (both estimated by ssGBLUP). For the growth traits of a 6-month-old, the trait with the largest heritability estimate under different matrices was BW (0.432), as estimated using ssGBLUP, whereas the trait with the smallest heritability estimate was BH (0.281), as estimated using PBLUP. The estimates of heritability for chest girth and CC estimated using PBLUP were higher than those estimated using ssGBLUP, whereas heritability estimated for the remaining traits using ssGBLUP was higher than that estimated using PBLUP. The standard errors of the heritability estimates were similar and low (0.030–0.103) for the different matrix heritability estimates, suggesting that the accuracy of the heritability estimates was consistent.

### 3.3. Genetic and Phenotypic Correlation

#### 3.3.1. Genetic and Phenotypic Correlation of Milk-Production Traits

**Figure 3:**
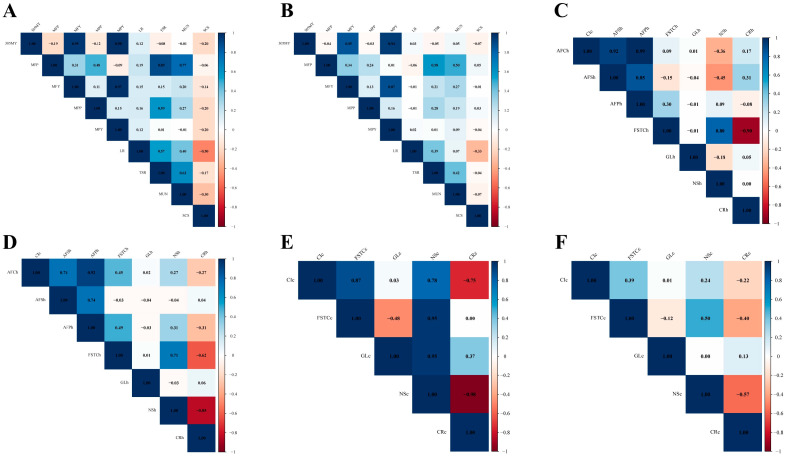
Genetic and phenotypic correlations of milk-production and reproduction traits. Genetic correlations (**A**,**C**,**E**); phenotypic correlations (**B**,**D**,**F**).

The genetic correlations between milk-production traits in the dual-purpose Simmental cattle ranged from −0.50 to 0.98 (Figure 3A). 305MY was highly genetically correlated with MFY and MPY; MFY was highly genetically correlated with MPY; MFP percentage was highly genetically correlated with TSR; TSR was moderately genetically correlated with MPP, LR, and MUN; and SCS was negatively genetically correlated with all other traits. Correlations were negative for the SCS and all other traits. The phenotypic correlation coefficients between the milk-production traits in the dual-purpose Simmental cows ranged from −0.33 to 0.94 (Figure 3B). 305MY was highly phenotypically correlated with MFY and MPY; MFY was highly phenotypically correlated with MPY; and MFP was moderately phenotypically correlated with TSR.

#### 3.3.2. Genetic and Phenotypic Correlation of Reproduction Traits

Genetic correlations between reproduction traits in the dual-purpose Simmental heifer cattle ranged from −0.90 to 0.99 (Figure 3C). AFCh was highly genetically correlated with AFS and AFP; AFS was highly genetically correlated with AFP; FSTCh was highly genetically correlated with NSh; and FSTCh was highly genetically correlated with CRh (negative). The phenotypic correlation coefficients between reproductive traits in heifer cows were from −0.85 to 0.92 (Figure 3D). AFCh showed a highly phenotypic correlation with AFPh; CRh showed a highly phenotypic correlation with NSh (negatively); AFCh showed a moderately phenotypic correlation with AFSh; FSTCh showed a moderately phenotypic correlation with NSh; and FSTCh showed a moderately phenotypic correlation with CRh (negative). The genetic correlations between the reproduction traits in the dual-purpose Simmental cattle ranged from −0.98 to 0.95 (Figure 3E). Except for GL vs. CI and FSTC vs. CR, which demonstrated low genetic correlation, a moderate-to-high genetic correlation was observed among all the traits. The phenotypic correlation coefficients between the reproductive traits in cows ranged from −0.57 to 0.50 (Figure 3F). FSTC was moderately phenotypically associated with NS, whereas NS was moderately phenotypically associated with CR (negative).

#### 3.3.3. Genetic and Phenotypic Correlation of Growth Traits

**Figure 4:**
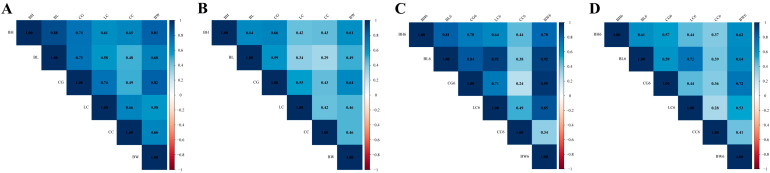
Genetic (**A**,**C**) and phenotypic correlations (**B**,**D**) of growth traits.

The genetic correlations between the newborn growth traits in the dual-purpose Simmental cattle ranged from 0.48 to 0.88 (Figure 4A). Medium-to-high genetic correlations were detected among all traits, except CC, which was moderately genetically correlated with BL and CG. The largest genetic correlation (0.88) was recorded between BH and BL. The phenotypic correlation between the newborn growth traits was 0.42–0.66 (Figure 4B). A moderate phenotypic correlation was noted between BH vs. BL and CG vs. BW, whereas a moderate phenotypic correlation was noted between CG vs. BL and LC vs. BW. The genetic correlations among growth traits at 6 months old ranged from 0.24 to 0.95 (Figure 4C). In addition, moderate-to-high genetic correlations were detected among all traits, except for low genetic correlations between CC vs. BH and BW and BL vs. LC. Phenotypic correlations among the growth traits at 6 months old ranged from 0.28 to 0.73 (Figure 4D). BH was moderately phenotypically correlated with other traits (except for LC and CC); BL was moderately phenotypically correlated with other traits (except CC); CG was moderately phenotypically correlated with BW; and LC and BW were moderately phenotypically correlated.

### 3.4. Reliability Prediction of Estimated Breeding Values (EBVs) and Genomic Estimated Breeding Values (GEBVs) Based on Different Matrices

#### 3.4.1. Reliability Comparison of the Breeding Values for Milk-Production Traits

**Figure 5:**
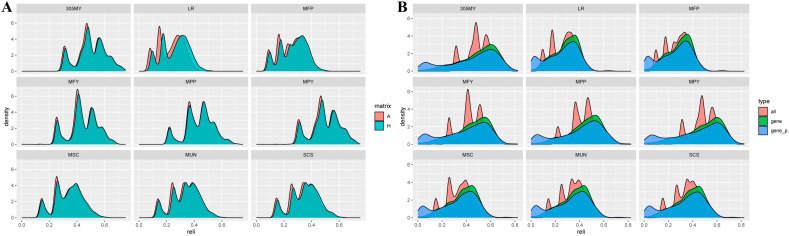
Density distribution plots of the reliability of breeding values for milk-production traits udder different matrices (**A**); evaluation of the reliability of breeding value results across different datasets using the H matrix (**B**).

Reliability and increased reliability of EBV and GEBV were noted in the whole and genotyped populations of dual-purpose Simmental cows for milk-production traits (Table 4). The reliability ranges were 0.233–0.496 and 0.265–0.506 for EBV and GEBV in the whole population and 0.275–0.549 and 0.215–0.566 for EBV and GEBV in the genotype population, respectively. Across different matrices, the reliability of 305MY remained the highest, with values of 0.549 and 0.566, respectively. The correlation coefficients between EBV and GEBV in the entire population were 0.958–0.990, with an increase in the reliability of GEBV of 0.6–3.2%; correlation coefficients between EBV and GEBV in the genotyped population were 0.873–0.966, with increased reliability of GEBV of 1.6–4%. The frequency distribution of the breeding value reliability for the milk-production trait displayed how the results based on the H-matrix were skewed to the right side, indicating a higher reliability (Figure 5A). The H-matrix enhancement of EBV reliability for each milk-production trait for the three datasets detected the smallest enhancement in reliability of breeding values for the data of all phenotyped individuals (red), but the enhancement of reliability for the genotyped individuals (green) and genotyped individuals and relatives (blue) did not show any significant difference, indicating that the H-matrix had a consistent reliability-enhancement effect on the latter two datasets (Figure 5B and Supplementary Figure S1).

#### 3.4.2. Reliability Comparison of the Breeding Values for Reproduction Traits

**Figure 6:**
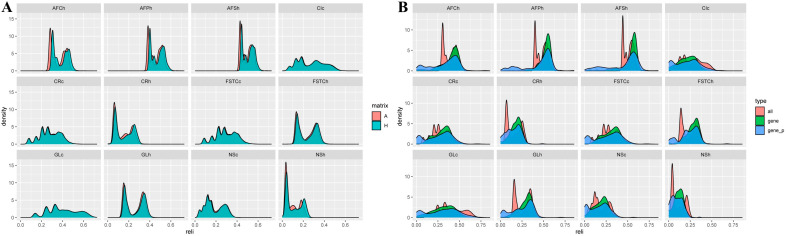
Density distribution plots of the reliability of breeding values for reproduction traits under different matrices (**A**); evaluation of the reliability of breeding value results across different datasets using the H matrix (**B**).

Reliability and increased reliability of EBV and GEBV in the whole and genotyped populations of dual-purpose Simmental cows for reproduction traits are shown in Table 5. The reliability ranges for EBV and GEBV in the entire population were 0.095–0.494 and 0.113–0.509, respectively, and those in the genotype population were 0.099–0.527 and 0.117–0.555, respectively. Across different matrices, the reliability of AFSh remained the highest, with values of 0.527 and 0.555, respectively. The correlation coefficients between EBV and GEBV in the whole population were 0.954–0.992, with an increase in the reliability of GEBV of 0.2–2.4%. In addition, the correlation coefficients between EBV and GEBV in the genotype population were 0.718–0.969, with an increase in the reliability of GEBV of 0.4–3.6%. The frequency distribution of the breeding value reliability for reproduction traits demonstrated that, except for some traits (such as CIc, CR, FSTC, GL, and NSc), the results based on the H-matrix were skewed toward the right side, indicating higher reliability (Figure 6A). The results of the H-matrix enhancement of EBV reliability for each of the breeding traits for the three data sets revealed the smallest enhancement of breeding value reliability for all phenotypic individual data (red), and the enhancement was located in the middle for genotypic individuals (green). The largest enhancement of reliability was detected for genotypic individuals and relatives (blue). A-matrix reliability enhancement was significant for some of the traits (e.g., CIc, GLc, and NSh) (Figure 6B and Supplementary Figure S2).

#### 3.4.3. Reliability Comparison of the Breeding Values for Growth Traits

**Figure 7:**
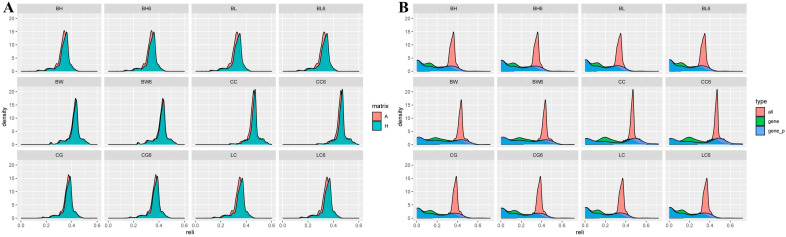
Density distribution plots of the reliability of breeding values for growth traits under different matrices (**A**); evaluation of the reliability of breeding value results across different datasets using the H matrix (**B**).

Reliability and increased reliability of EBV and GEBV in the whole and genotyped populations of dual-purpose Simmental cows for growth traits are shown in Table 6. The reliability ranges for EBV and GEBV were 0.275–0.451 and 0.275–0.463 in the whole population and 0.242–0.433 and 0.158–0.427 for EBV and GEBV, respectively, in the genotype population. The correlation coefficients between EBV and GEBV in the whole population were 0.872–0.998, showing an increase in the reliability of GEBV of 0.5–1.5%; the correlation coefficients between EBV and GEBV in the genotypic population were 0.819–0.964, with a decrease in the reliability of GEBV of 12–17%. The frequency distribution of the breeding-value reliability for the growth trait revealed that the results based on the H-matrix were skewed to the right side, indicating higher reliability (Figure 7A). The results of the H-matrix on the improvement of EBV reliability for each growth trait for the three datasets detected the largest improvement in the reliability of breeding values for all phenotypic individual data (red), with the smallest improvement for genotyped individuals (green). The results for genotyped individuals and relatives (blue) were located in the middle of the range (Figure 7B and Supplementary Figure S3).

## 4. Discussion

### 4.1. Genetic Parameters of Traits in Dual-Purpose Simmental Cattle

In the actual breeding work, when considering only the selection of a trait during the selection of breeding animals, the trait showed faster improvement. However, due to the existence of a genetic correlation and a negative genetic correlation between the traits, “negative progress” was noted. Therefore, multiple economically important traits with direct, indirect, or potential economic benefits should be considered during the development of breeding programs, so as to achieve balanced breeding. The genetic evaluation of economically important traits can provide a basis for the selection of target traits. Genetic and environmental factors can affect the growth traits of cattle, and heritability plays an important role in phenotypic individual differences [24].

#### 4.1.1. Heritability of the Milk-Production Traits

The 305MY, MFP, MFY, MPP, MPY, and SCS of the dual-purpose Simmental cattle were 4753.61 kg, 3.94%, 183.55 kg, 3.51%, 161.87 kg, and 2.23, respectively, which were comparable to the corresponding values for the dual-purpose Simmental cattle population (305MY-5469.05 kg, MFP-4.13%, MPP-3.33%, and SCS-4.23) [25], the Xinjiang brown cattle population (305MY-4126.49 kg, MFP-3.93%, MFY-168.53 kg, MPP-3.37%, MPY-143.71 kg, and 4.98) [26,27], and New Zealand dairy cows (305MY-4969 kg, MFP-4.75%, MFY-232 kg, MPP-3.83%, MPY-188 kg) [28]. Dual-purpose Simmental cattle produce less milk overall when compared to dairy cows (MY-9807.07 kg, MFP-3.54, and MPP-3.19), but they have higher MFP and MPP [29]. Owing to the functional differences among the studied breeds, energy gained by dual-purpose cows is required to maintain basal metabolism, muscle development, milk synthesis, and reproduction, in addition to providing energy for meat performance.

In this study, we estimated the heritability estimates for milk-production traits using a single-trait model, based on PBLUP and ssGBLUP, and found it to be of moderate heritability level (except MFP and MPP). Based on PBLUP for genetic parameter estimation, Wei et al. [25] found that the heritability values were 0.22 for 305MY, 0.13 for MFP, 0.10 for MPP, and 0.03 for SCS in a dual-purpose Simmental cow population. Zhou et al. [26] and Zhang et al. [27] reported heritability values of 0.24–0.40 for 305MY, 0.08 for MFP, 0.07–0.30 for MFY, 0.30 for MPP, 0.14–0.20 for MPY, and 0.04–0.08 for SCS in a Xinjiang brown cow population. Based on GBLUP and ssGBLUP for genetic parameter estimation, Wolf et al. [30] reported heritability of 0.41–0.43, 0.54–0.56, 0.34–0.36, 0.62–0.65, 0.35–0.37, and 0.09–0.10 for dual-purpose German Black Pied cattle. Laodim, et al. [31] found that the heritability values were 0.25 and 0.18 for 305MY and MFY in a Thai multibreed dairy population. The results of this study for 305MY (0.31), MFP (0.09), MFY (0.25), and MPP (0.22) fall between the estimates reported in the above-mentioned research reports, while the results for MPY (0.31) and SCS (0.15) are higher than those reported in the above-mentioned research reports. Compared with other studies that estimate genetic parameters based on PBLUP, this study heritability results of 305MY, MFP, MFY, MPP, and MPY were higher than the reported corresponding values of 0.07–0.30, 0.12–0.34, 0.23, 0.20–0.38, and 0.23 for the dairy cow population [29,32,33].

#### 4.1.2. Heritability of the Reproduction Traits

In the dual-purpose breed study, Simmental cows had CIs of 380–430 days for 1–5 parity and gestation lengths of 282–284 days for 1–6 parity [34]. AFC, AFS, FSTCh, GLh, NSh, CI, FSTCc, GLc, and NSc results for Xinjiang brown cattle have been reported as 30 months of age, 20 months of age, 6.85 days, 283.90 days, 1.17 times, 429.44 days, 13.18 days, 284.86 days, and 1.37 times, respectively [26,35,36]. AFP, AFC, GLc, and CIc results for Three Rivers Cattle have been reported as 23 months of age, 31 months of age, 280.61 days, and 383.04 days, respectively [37]. In this study, the phenotypic values of AFC, AFS, GLh, CI, GLc, NSh, and NSc were similar to the results reported earlier for the dual-purpose cattle. Phenotypic values of FSTCh and FSTCc were higher than the corresponding results for the dual-purpose cattle, but phenotypic values of AFP were lower than the results for Sanhe cattle. The phenotypic values of AFC (30 months) were higher than 26.7 months in Holstein cows [38], 25 months in Hanwoo cows and Israeli dairy cattle [39,40], <34 months in Jersey crossbred cattle [41], 35.24 months in Nellore cows [42], and 34.32 months in Retinta beef cattle [43]. The dual-purpose Simmental cattle phenotypic values of CI were comparable to 12.4–12.6 months obtained for Hanwoo Cows [39], although these values were higher than 12.3 months reported for dairy cows [28]. Early AFC may be associated with inadequate mammary gland development or an increased risk of difficult deliveries, and late AFC or a long CI can increase the feeding costs, including feed and labor. Malnutrition, infections, anestrus, long-term infertility, frozen semen, and other factors can increase disorders related to reproduction incidence. Therefore, the following aspects need to be strengthened in ranch management: (1) optimization of concentrated feed formulation, maintenance of cattle body condition, and prevention of reproductive disorders caused by malnutrition; (2) enhancement of training management for breeding technicians on ranch management software and artificial insemination procedures to reduce breeding failures caused by non-standard operations, minimization of external environmental impacts on reproductive traits, and establishment of a more accurate data foundation for genetic parameter estimation; and (3) enhancement of pasture hygiene management (e.g., by increasing manure removal frequency and bedding replacement).

We estimated that the heritability estimates for reproduction traits using a single-trait model, based on PBLUP and ssGBLUP, were of low heritability level (except AFCh, AFSh, and AFPh). Based on PBLUP for genetic parameter estimation, as previously reported, the heritability of AFC, AFS, FSTCh, GLh, NSh, CI, FSTCc, GLc, and NSc were 0.01–0.17, 0.01–0.04, 0.34, 0.001, 0.39, 0.0048–0.08, 0.01, 0.009–0.07, and 0.06 in Xinjiang brown cattle [26,35,36]. Dong et al. [37], using the PBLUP models analysis, reported that the heritability values were 0.06 for AFC, 0.08 for CI, and 0.05 for GLc in the Sanhe cow population. Canaza-Cayo et al. [44] reported a heritability of 0.18 for AFC in Brazilian dual-purpose Girolando cattle. The CI heritability in the current study was consistent with the abovementioned reported results. The heritability of AFC, AFS, AFP, GLh, NSh, FSTCc, GLc, and NSc was greater than that of the abovementioned results, whereas the heritability of FSTCh was less than that of the Xinjiang brown cattle. When compared with other breeds, the heritability of AFS in this study was in agreement with 0.48 in Chinese Holstein cattle [45] and higher than the heritability of 0.02–0.13 in other dairy cattle populations [38,46]. Past studies on beef cattle populations, such as Canchim beef cattle, Hanwoo cows, Nellore cows, and Retinta beef cattle using the PBLUP models reported a heritability of AFC of 0.04, 0.07–0.137, 0.11–0.165, and 0.223 [43,46,47,48,49]; for dairy cattle populations, the heritability of AFC was 0.08, 0.170, 0.231, and 0.247 [38,46,50], which was lower than the present results. Additionally, Kluska, et al. [51] analyzed Nellore cattle using the ssGBLUP model and reported a heritability of 0.20 for AFC, which is lower than the findings of this study. Based on PBLUP for genetic parameter estimation, the heritability of GL in dual-purpose Simmental cows (0.15) was higher than that in Canadian Jersey cows (0.08–0.11) [52], lower than that in Chinese Holstein cows (0.22) [45], French Montbéliard (0.58), French Simmental cows (0.56), and French Swiss Brown cows (0.49) [53], and similar to the results for Hanwoo cattle, of 0.10–0.16 [39,49]. The CI heritability in the present study using PBLUP was similar to the results for Hanwoo cattle, of 0.01–0.07 [39,49]. In this study, the heritability of NS was estimated to be 0.032–0.044 using the PBLUP model and 0.039–0.042 using the ssGBLUP model, both of which were higher than the heritability of Chinese Holstein cattle (0.015–0.027/0.018–0.031) [54], and lower than that in Thai-Holstein crossbreeds (0.038/0.051) [55]. Using PBLUP and ssGBLUP models, the reproductive traits showed low heritability in studies on various breeds and were affected by multiple factors in genetic and biological complexity, as well as by difficulty and limited accuracy of the measurement. In addition, because of the natural selection for health-related traits, fertility mutations or variations are maintained at a low frequency, which yields low genetic variation and heritability for reproduction-related traits [56]. However, inter- and intra-breed variation in fecundity implies a potential genetic variation, and the genetic composition of fecundity is altered and reduces the genetic progression of milk-production traits [57].

#### 4.1.3. Heritability of the Growth Traits

The comparison of growth traits and phenotypic values among the varieties is shown in Table S7 [58,59,60,61,62,63,64,65]. In this study, the mean values of the growth traits were consistent with those of the dual-purpose Simmental cattle. The BW aligned with relevant findings for Chinese Simmental beef cattle, while the BH and BL at 6 months of age were higher than those reported in Chinese Simmental beef cattle. Dual-purpose Simmental cattle and beef Simmental cattle differed in their breeding objectives. The former aims for balanced improvement in both milk and meat production, while the latter prioritizes growth rate and carcass performance development. The approach to nutrient management was different in these cases, with the beef Simmental cattle opting for a more efficient fattening method. The feeding regimens during calfhood (i.e., suckling, suckling + feeding stage) also influenced the development of divergent growth performance characteristics. The growth traits of the dual-purpose Simmental cattle were in the middle level relative to those of the Xinjiang brown cattle, Sanhe cattle, Angus cattle, Jinnan cattle, and Brahman cattle. The genetic background, breeding direction, environmental adaptability, and feeding management style (such as housed or grazing) affected the phenotypic values of the growth traits. Previous studies also reinforced the importance of growth data collection from dual-purpose herds [66,67].

We estimated the heritability of growth traits using a single-trait model, based on PBLUP and ssGBLUP approaches, finding them to exhibit low-to-moderate heritability levels. Ren et al. [63] reported that heritability estimates for birth BH, BL, CG, CC, and BW in Sanhe cattle were 0.46, 0.48, 0.38, 0.50, and 0.41, respectively. Similarly, Xu et al. [64] found that the heritability of BH, BL, CG, and BW at birth (and at 6 months of age) in Xinjiang brown cattle was 0.46 (0.43), 0.34 (0.40), 0.61 (0.35), and 0.30 (0.22), respectively. Guo et al. [60] reported corresponding heritability estimates in Jinnan cattle for birth (and 6 months of age) BH, BL, CG, CC, and BW as 0.28 (0.23), 0.20 (0.15), 0.26 (0.24), 0.41 (0.20), and 0.26 (0.23), respectively. Quantitative traits, as important economic traits, are susceptible to genetic and environmental influences; growth traits during the newborn stage are primarily influenced by maternal effects and additive genetic effects, while with increasing age, growth traits become more affected by various environmental factors such as feeding management and nutrition. Therefore, conducting stage genetic parameter estimation for growth traits allows for selecting appropriate fixed effects and models based on the data characteristics of each stage, clarifying genetic relationships across stages, and providing an accurate data foundation for defining breeding objectives and selection strategies. Environmental factors are an important consideration that cannot be ignored in livestock breeding work. In addition to the fixed effects already considered in this study (e.g., herd, year, season, sex, parity and inseminator), there are other effects exist, such as dam age, birth type (e.g., single, twin or multiple), calving type (e.g., unassisted, dystocia or assistance), calving number, sire and interactions between factors. On the one hand, effectively analyzing the impact of non-genetic factors on various traits can improve the accuracy of genetic evaluation; on the other hand, reasonable and effective management measures can be formulated based on the impact of different effects, to minimize the effects caused by environmental variation.

### 4.2. Genetic and Phenotypic Correlations for Dual-Purpose Simmental Cattle

Recently, several countries have begun to focus more on balanced breeding in their cattle-breeding efforts by adjusting the selection and relative weights of target traits in the selection indices. For example, the Unique Synthesis Index (ISU) of the French Montbéliard Beef Association increased the importance of cannon girth and chest depth from 10% to 35%, the importance of udder health traits (such as SCS and mastitis) has increased from 12.5% to 14.5%, and the reproduction traits have increased from 12.5% to 18%. The Gesamt ZuchtWert for German Simmental cows has increased the relative economic weight of MFY from 4.5% to 18.6%, decreased the relative economic weight of MPY from 33.4% to 19.4%, and increased the reproductive traits from 6.8% to 14%. It is therefore important to harmonize the development of dairy and meat performance and maximize the economic benefits. An association was noted among quantitative traits, wherein the selection of a trait indirectly influenced other characteristics [68].

The current study found −0.50 to 0.98 and −0.33 to 0.94 genetic and phenotypic correlations for milk-production traits in dual-purpose Simmental cows, respectively. Several studies have demonstrated that 305MY in dual-purpose Simmental, Xinjiang Brown, Italian Swiss Brown, and dairy cows was negatively correlated with MFP, MPP, and SCS (genetic correlation 0.02 to 0.65/phenotypic correlation 0.01–0.43) [25,36,69,70]. The present results are consistent with those from the abovementioned studies. In addition, MY was highly positively correlated with both MFY and MPY in the present study, as well as in the literature [70,71,72,73]. As such, the higher the milk yield, the lower the milk fat rate and the milk protein rate. The milk fat rate is significantly affected by nutrition and management, but the milk protein rate is significantly affected by genetics; therefore, in actual production, genetic selection, nutrition (dietary concentrate/coarse ratio, etc.), and feeding management strategies are employed to achieve a balance among the three factors. Another important indicator is the somatic cell count (SCC), which is closely related to milk yield and milk quality. For instance, elevated SCC decreases the casein and milk fat content. However, the occurrence of mastitis reduces milk yield and increases feeding costs. Therefore, it is necessary to focus on the cleanliness of the feeding environment and the standardization of the milking procedure to stabilize and improve the efficiency of the farm.

Genetic correlations between fertility and production traits were found to be negative in past studies (range 0.35–0.60), while fertility was reduced due to high selection for production traits [74]. However, nowadays, breeding techniques are becoming increasingly sophisticated and well-managed [75,76], and the selection of reproductive traits in breeding is gaining attention. The current study recorded −0.98 to 0.99 and −0.85 to 0.92 genetic and phenotypic correlations for reproduction traits in dual-purpose Simmental cows, respectively. Moreover, AFC was positively correlated with the remaining reproductive traits, but negatively correlated with the number of repeated matings. Meanwhile, CI was positively correlated with the number of repeated matings, implying that younger age at first calving and shorter calving intervals result in fewer matings, which indirectly reduces the number of frozen semen used, thereby reducing costs.

In this study, we identified positive genetic (0.44–0.88/0.24–0.92) and phenotypic (0.29–0.66/0.28–0.73) correlations between growth traits in Simmental cattle at identical stages. The TPI in China includes birth weight, weight at 6 months of age, and weight at 18 months of age. The current formula for estimating the cow’s BW includes both CG and BL (BW = CG^2^ × BL × 90). The chest width reflects an individual’s ability to produce high yields, as well as the durability of maintaining high yields. In this study, newborn weight and weight at 6 months of age were both highly correlated with body size traits at the same stage. The next highest genetic correlation of BW with BL and CG was 0.68 (0.92) and 0.82 (0.95), respectively; the highest phenotypic correlation of BW with BL and CG was 0.64 (0.61) and 0.66 (0.57), respectively. The genetic (phenotypic) correlation coefficients for BW vs. BH and CG were comparable to 0.11–0.78 (0.13–0.67), 0.35–0.92 (0.50–0.81) obtained for the Xinjiang brown cattle [64] and 0.86 (0.42) and 0.56 (0.50) obtained for the Sanhe cattle [63]. In a previous study, the birth weight of Simmental female calves was significantly correlated with the chest circumference at 6 months of age (0.30), while the weight at 6 months of age was highly significantly correlated with the body slant length and chest circumference (0.58, 0.81) [65]. Therefore, balancing the relationships among traits in breeding efforts by employing comprehensive selection indices to rank pairs of frozen semen and in-herd cows, as well as the development of breeding and mating programs on the ranch, in conjunction with population-inbreeding coefficients, can yield greater genetic progress and benefits.

### 4.3. Reliability of EBV and GEBV for Each Trait in Dual-Purpose Simmental Cattle

In the whole population, prediction reliabilities for milk-production, reproduction, and growth traits were 0.233–0.496, 0.095–0.494, and 0.275–0.451 using PBLUP models, while ssGBLUP models achieved 0.265–0.506, 0.113–0.509, and 0.275–0.463; the ssGBLUP model was superior to the BLUP model. In the genotyped subpopulation, prediction reliabilities for milk-production, reproduction, and growth traits were 0.275–0.549, 0.099–0.527, and 0.242–0.433 using PBLUP models, while ssGBLUP models achieved 0.215–0.566, 0.117–0.555, and 0.158–0.427; the ssGBLUP model was superior to the BLUP model. Zhang et al. [27] studied milk-production in a Xinjiang Brown cattle whole population and found predictive accuracy for the BLUP and ssGBLUP models to be 0.45–0.75 and 0.52–0.85, respectively, while the breeding value reliabilities in the genotyped subpopulation were 0.161–0.491 and 0.172–0.526; the ssGBLUP model was superior to the BLUP model. Naserkheil et al. [77] studied carcass traits in a Korean cattle population and found mean values of predictive accuracy for the BLUP and ssGBLUP models to be 0.45–0.75 and 0.52–0.85, respectively; they noted that the ssGBLUP model was superior to the BLUP model. Peripolli et al. [78] reported that the ssGBLUP model predicted reproduction traits in Nelore beef cattle with 25.6% more accuracy than that in the BLUP model. Combining these results showed that the prediction was more accurate for traits with a higher heritability [79]. However, for low-heritability traits, such as reproduction, the ssGBLUP model can be used to improve the accuracy of estimation [46,55]. In addition to heritability, the reference population size affects the accuracy of genomic prediction, based on the results of the integration of four European Holstein populations, showing improved reliability of different traits by 2–19% [80]. Moreover, the addition of the Nordic and French Holstein populations to the Brazilian Holstein population also improves the accuracy of genomic prediction [81]. In addition, it has been shown that combining Chinese Holstein cattle populations with Xinjiang brown cattle populations to form a joint reference group has a positive effect on the genome prediction of Xinjiang brown cattle [82]. After preliminary evaluation of the genetic structure and genetic relationship among various populations through principal component analysis, phylogenetic analysis, population ancestry component analysis, and linkage disequilibrium analysis, the populations genetically linked with that of this study population into one large reference population, which can improve the accuracy of genomic prediction of dual-purpose Simmental cattle.

## 5. Conclusions

The milk-production traits in dual-purpose Simmental cows were of low-to-moderate heritability, the reproductive traits in heifers were of moderate-to-high heritability (except for NSh, which is of low heritability), and the reproductive traits in cows were of low-to-moderate heritability, whereas the growth traits were of moderate-to-high heritability. The using the H-matrix enhances the reliability of traditional genetic evaluation EBV to varying degrees. Constructing a multi-trait model incorporating milk production, reproduction, and growth traits is recommended to facilitate early, multi-objective selection of breeding stock, thereby shortening the breeding cycle. The ssGBLUP method improves the accuracy of breeding value estimation. Subsequent genome-wide association studies can be utilized to identify significant single nucleotide polymorphisms for further optimization of the genomic selection model and enhancement of prediction accuracy. Subsequent increasing the size of the reference population will contribute to enhancing the reliability of EBV for milk production, reproduction, and growth traits, and serve as part of an early selection strategy to predict the breeding value of young bulls, thereby reducing breeding costs.

## Figures and Tables

**Table 1:** Significance test results of variance for milk-production traits (*F* value).

Trait	Herd	Parity	Calving Year	Calving Season	Lactation
305MY	1923.31 **	2.31 *	10.90 **	2.94 *	-
MFP	1157.58 **	14.18 **	6.62 **	103.52 **	97.97 **
MFY	1745.86 **	3.02 *	36.99 **	0.03 *	-
MPP	18.99 **	7.84 **	86.97 **	74.86 **	5.22 **
MPY	1326.81 **	4.66 **	13.79 **	5.31 **	-
LR	132.82 **	63.76 **	39.14 **	1.32 ^ns^	39.57 **
TSR	50.37 **	15.57 **	64.13 **	6.39 **	2.18 ^ns^
MUN	2533.45 **	4.77 **	206.25 **	139.86 **	17.08 **
SCS	1263.25 **	5.52 **	18.31 **	2.10 ^ns^	9.91 **

Note: 305MY: 305 daily milk yield; MFP: milk fat percentage; MFY: milk fat yield; MPP: milk protein percentage; MPY: milk protein yield; LP: lactose percentage; TSR: total solids rate; MUN: milk urea nitrogen; SCS: somatic cell score; **: extremely significant effect (*p* < 0.01); *: significant effect (*p* < 0.05); ns: no significant effect (*p* > 0.05).

**Table 2:** Significance test results of variance for reproduction traits (*F* value).

Trait	Herd	Breeding Technician		Service Year		Service Season		Parity
		First	Last	First	Last	First	Last	
AFCh	-	-	69.28 **	-	-	60.63 **	5.53 **	-
AFSh	518.64 **	-	-	547.84 **	74.18 **	-	-	-
AFPh	-	-	29.26 **	-	-	103.03 **	1.10 ^ns^	-
FSTCh	-	-	35.24 **	-	-	27.80 **	7.18 **	-
GLh	267.56 **	-	-	-	-	38.75 **	34.19 **	-
NSh	-	18.71 **	-	8.71 **	0.78 ^ns^	-	-	-
CRh	-	25.64 **	-	7.13 **	0.63 ^ns^	-	-	-
CIc	656.62 **	-	-	-	-	5.27 **	0.25 ^ns^	15.64 **
FSTCc	-	-	68.61 **	-	-	19.80 **	20.08 **	10.23 **
GLc	687.59 **	-		-	-	67.72 **	91.00 **	4.51 **
NSc	-	-	23.64 **	-	-	31.46 **	38.00 **	12.12 **
CRc	-	22.30 **	16.59 **	13.41 **	-	-	-	16.78 **

Note: AFCh: age at first calving in heifer; AFSh: age at first service in heifer; AFPh: age at first pregnancy in heifer; FSTCh: interval from first service to conception in heifer; GLh: gestation length in heifer; NSh: number of services in heifer; CRh: conception rate for first service in heifer; CIc: calving interval in cow; FSTCc: interval from first service to conception in cow; GLc: gestation length in cow; NSc: number of services in cow; CRc: conception rate for first service in cow. **: extremely significant effect (*p* < 0.01); ns: no significant effect (*p* > 0.05).

**Table 3:** Significance test results of variance for growth traits (*F* value).

Trait	Newborn			Six-Month-Old		
	Birth Year	Birth Month	Sex	Birth Year	Birth Month	Sex
BH	52.99 **	1.42 ^ns^	119.12 **	85.71 **	6.21 **	12.49 **
BL	88.93 **	1.79 ^ns^	83.85 **	167.47 **	13.30 **	25.09 **
CG	22.61 **	2.74 **	129.63 **	193.72 **	4.81 **	26.40 **
LC	32.03 **	3.23 **	93.85 **	74.47 **	1.55 **	4.26 **
CC	42.34 **	5.90 **	181.18 **	176.00 **	21.12 **	176.48 **
BW	13.04 **	2.30 **	351.82 **	165.55 **	8.77 **	28.60 **

Note: BH: body height; BL: body length; CG: chest girth; LC: leg circumference; CC: cannon circumference; BW: body weight. **: extremely significant effect (*p* < 0.01); ns: no significant effect (*p* > 0.05).

**Table 4:** Comparison of EBV reliability, GEBV reliability, and reliability gain (∆rel) for milk-production traits between the whole population and the genotyped population.

Traits	Whole Population				Genotyped Subpopulation			
	PBLUP	ssGBLUP	∆rel (%)	Correlation	PBLUP	ssGBLUP	∆rel (%)	Correlation
305MY	0.496	0.506	1	0.989 **	0.549	0.566	1.7	0.966 **
MFP	0.254	0.271	1.7	0.975 **	0.294	0.319	2.5	0.881 **
MFY	0.440	0.450	1	0.985 **	0.487	0.505	1.8	0.957 **
MPP	0.432	0.438	0.6	0.986 **	0.480	0.496	1.6	0.945 **
MPY	0.491	0.502	1.1	0.990 **	0.538	0.557	1.9	0.965 **
LR	0.233	0.265	3.2	0.958 **	0.275	0.315	4	0.846 **
TSR	0.332	0.341	0.9	0.979 **	0.387	0.404	1.7	0.916 **
MUN	0.329	0.342	1.3	0.973 **	0.375	0.397	2.2	0.873 **
SCS	0.339	0.354	1.5	0.974 **	0.389	0.413	2.4	0.909 **

Note: 305MY: 305 daily milk yield; MFP: milk fat percentage; MFY: milk fat yield; MPP: milk protein percentage; MPY: milk protein yield; LP: lactose percentage; TSR: total solids rate; MUN: milk urea nitrogen; SCS: somatic cell score; **: extremely significant effect (*p* < 0.01).

**Table 5:** Comparison of EBV reliability, GEBV reliability, and reliability gain (∆rel) for reproduction traits between the whole population and the genotyped population.

Traits	Whole Population				Genotyped Subpopulation			
	PBLUP	ssGBLUP	∆rel (%)	Correlation	PBLUP	ssGBLUP	∆rel (%)	Correlation
AFCh	0.359	0.383	2.4	0.986 **	0.393	0.429	3.6	0.929 **
AFSh	0.494	0.509	1.5	0.991 **	0.527	0.555	2.8	0.969 **
AFPh	0.456	0.474	1.8	0.992 **	0.503	0.534	3.1	0.945 **
FSTCh	0.233	0.244	1.1	0.975 **	0.268	0.287	1.9	0.882 **
GLh	0.251	0.262	1.1	0.983 **	0.280	0.303	2.3	0.902 **
NSh	0.095	0.113	1.8	0.954 **	0.099	0.117	1.8	0.718 **
CRh	0.143	0.157	1.4	0.970 **	0.163	0.181	1.8	0.820 **
CIc	0.275	0.275	0	0.971 **	0.242	0.254	1.2	0.814 **
FSTCc	0.293	0.292	−0.1	0.979 **	0.313	0.325	1.2	0.895 **
GLc	0.398	0.400	0.2	0.989 **	0.359	0.377	1.8	0.889 **
NSc	0.186	0.185	−0.1	0.978 **	0.199	0.203	0.4	0.882 **
CRc	0.280	0.280	0	0.987 **	0.301	0.313	1.2	0.883 **

Note: AFCh: age at first calving in heifer; AFSh: age at first service in heifer; AFPh: age at first pregnancy in heifer; FSTCh: interval from first service to conception in heifer; GLh: gestation length in heifer; NSh: number of services in heifer; CRh: conception rate for first service in heifer; CIc: calving interval in cow; FSTCc: interval from first service to conception in cow; GLc: gestation length in cow; NSc: number of services in cow; CRc: conception rate for first service in cow; **: extremely significant effect (*p* < 0.01).

**Table 6:** Comparison of EBV reliability, GEBV reliability, and reliability gain (∆rel) for growth traits between the whole population and the genotyped population.

Traits	Whole Population				Genotyped Subpopulation			
	PBLUP	ssGBLUP	∆rel (%)	Correlation	PBLUP	ssGBLUP	∆rel (%)	Correlation
Newborn								
BH	0.325	0.338	1.3	0.988 **	0.302	0.164	−13.8	0.842 **
BL	0.310	0.325	1.5	0.987 **	0.289	0.158	−13.1	0.857 **
CG	0.361	0.369	0.8	0.996 **	0.337	0.179	−15.8	0.891 **
LC	0.334	0.349	1.5	0.994 **	0.313	0.169	−14.4	0.909 **
CC	0.451	0.463	1.2	0.996 **	0.433	0.262	−17.1	0.909 **
BW	0.415	0.420	0.5	0.996 **	0.351	0.222	−12.9	0.916 **
Six months								
BH	0.325	0.338	1.3	0.978 **	0.302	0.164	−13.8	0.906 **
BL	0.310	0.325	1.5	0.974 **	0.289	0.158	−13.1	0.922 **
CG	0.361	0.369	0.8	0.981 **	0.337	0.179	−15.8	0.915 **
LC	0.334	0.349	1.5	0.998 **	0.313	0.169	−14.4	0.964 **
CC	0.451	0.463	1.2	0.973 **	0.433	0.262	−17.1	0.916 **
BW	0.415	0.420	0.5	0.986 **	0.351	0.222	−12.9	0.944 **

Note: BH: body height; BL: body length; CG: chest girth; LC: leg circumference; CC: cannon circumference; BW: body weight; **: extremely significant effect (*p* < 0.01).

## Data Availability

The data analyzed in this study is subject to the following licenses/restrictions: This manuscript utilizes proprietary data. Requests to access these datasets should be directed to the corresponding author, X.H., au-huangxixia@163.com.

## Supplementary Materials

The following supporting information can be downloaded at: https://www.mdpi.com/article/10.3390/ani15131960/s1, Figure S1. Density distribution plot of breeding value reliability for milk-production traits under different matrices. (A) Genotyped populations; (B) Genotyped populations and kinship groups. Figure S2. Density distribution plot of breeding value reliability for reproduction traits under different matrices. (A) Genotyped populations; (B) Genotyped populations and kinship groups. Figure S3. Density distribution plot of breeding value reliability for growth traits under different matrices. (A) Genotyped populations; (B) Genotyped populations and kinship groups. Table S1. Descriptive statistics of the milk-production traits of dual-purpose Simmental cattle. Table S2. Descriptive statistics of the reproduction traits of dual-purpose Simmental cattle. Table S3. Descriptive statistics of the growth traits of dual-purpose Simmental cattle. Table S4. Estimates of additive genetic variance, residual variance, and heritability for milk-production traits in dual-purpose Simmental cattle. Table S5. Estimates of additive genetic variance, residual variance, and heritability for reproduction traits in dual-purpose Simmental cattle. Table S6. Estimates of additive genetic variance, residual variance, and heritability for growth traits in dual-purpose Simmental cattle. Table S7. Comparison of growth traits among breeds.
