# Supplementary material for: Genomic Selection for Economically Important Traits in Dual-Purpose Simmental Cattle
Source: Animals (Basel). 2025 Jul 3;15(13):1960. doi: 10.3390/ani15131960 (PMC12249443; doi:10.3390/ani15131960)
Supplement: Supplementary file 1 [file animals-15-01960-s001.zip › Figure S1-S3 Density distribution plot of breeding value reliability for milk production, reproduction and growth traits under different matrices.pdf]

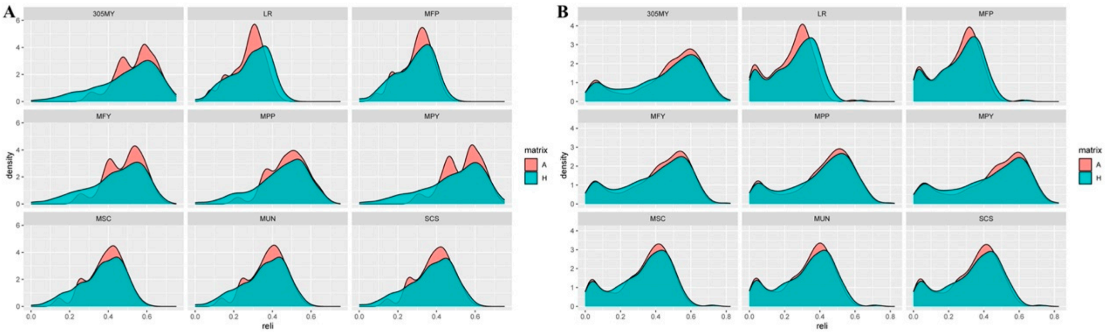

Figure S1. Density distribution plot of breeding value reliability for milk production traits under different matrices. (A) Genotyped populations; (B) Genotyped populations and kinship groups.

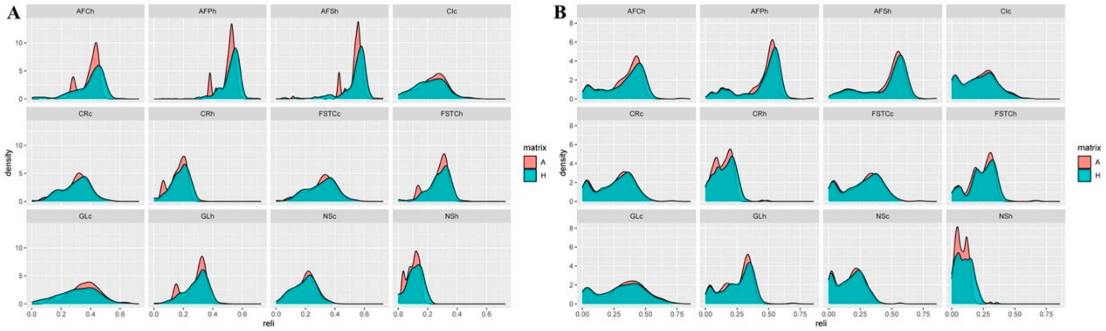

Figure S2. Density distribution plot of breeding value reliability for reproduction traits under different matrices. (A) Genotyped populations; (B) Genotyped populations and kinship groups.

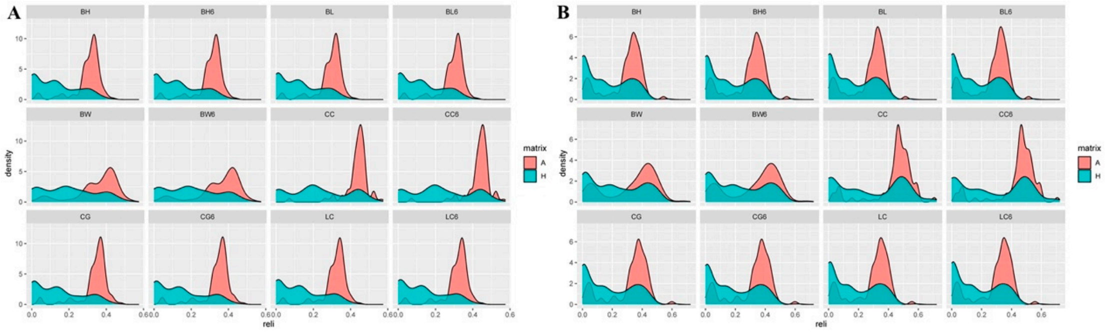

Figure S3. Density distribution plot of breeding value reliability for growth traits under different matrices. (A) Genotyped populations; (B) Genotyped populations and kinship groups.
