# Supplementary material for: Genomic Selection for Economically Important Traits in Dual-Purpose Simmental Cattle
Source: Animals (Basel). 2025 Jul 3;15(13):1960. doi: 10.3390/ani15131960 (PMC12249443; doi:10.3390/ani15131960)
Supplement: Supplementary file 1 [file animals-15-01960-s001.zip › Table S1-S3. Summary statistic for milk-production, reproduction and growth traits in dual-purpose Simmental population.pdf]

Table S1. Summary statistic for milk-production traits in dual-purpose Simmental population.

Traits	Number	Mean	SD	Min	Max	CV(%)
305MY (kg)	6214	4753.61	2088.59	1502.00	11598.00	43.94
MFP (%)	8049	3.94	0.85	1.51	6.99	21.57
MFY (kg)	5768	183.55	92.15	28.22	778.15	50.20
MPP (%)	8191	3.51	0.32	2.00	4.92	9.12
MPY (kg)	5822	161.87	68.06	43.41	488.23	42.05
LP (%)	7586	5.00	0.29	3.83	5.70	5.80
TSR (%)	7654	12.96	1.11	9.08	16.70	8.56
MUN (mg/dl)	8171	15.00	4.04	2.50	27.40	26.93
SCS	8009	2.23	1.93	-2.64	10.63	86.55

Note: 305MY: 305 daily milk yield; MFP: milk fat percentage; MFY: milk fat yield; MPP: milk protein percentage; MPY: milk protein yield; LP: lactose percentage; TSR: total solids rate; MUN: milk urea nitrogen; SCS: somatic cell score; CV: coefficient of variation.

Table S2. Summary statistic for reproduction traits in dual-purpose Simmental population.

Traits	Number	Mean	SD	Min	Max	CV(%)
AFCh (d)	5306	887.78	163.26	515	1222	18.39
AFSh (d)	5777	567.91	147.40	277	900	25.95
AFPh (d)	6164	624.65	185.44	255	1260	29.69
FSTCh (d)	7927	34.78	67.04	1	366	-
GLh (d)	7430	283.35	7.38	253	309	2.60
NSh	7321	1.55	0.85	1	4	54.84
CRh	8106	1.58	0.49	0	1	31.01
CIc (d)	13776	390.90	72.82	270	700	18.63
FSTCc (d)	16282	36.88	61.85	1	366	-
GLc (d)	13680	284.92	6.81	260	309	2.39
NSc	15120	1.85	1.13	1	5	61.08
CRc	16823	1.48	0.50	0	1	33.78

Note: AFCh: Age at first calving in heifer; AFSh: Age at first service in heifer; AFPh: Age at first pregnancy in heifer; FSTCh: Interval from first service to conception in heifer; GLh: Gestation length in heifer; NSh: Number of services in heifer; CRh: Conception rate for first service in heifer; CIc: Calving interval in cow; FSTCc: Interval from first service to conception in cow; GLc: Gestation length in cow; NSc: Number of services in cow; CRc: Conception rate for first service in cow; CV: coefficient of variation.

Table S3. Summary statistic for growth traits in dual-purpose Simmental population.

Stage	Trait	Number	Mean	SD	Min	Max	CV(%)
newborn	BH (cm)	4190	76.72	3.15	66.00	86.00	4.11
	BL (cm)	4099	75.08	3.61	65.00	86.00	4.81
	CG (cm)	4187	78.79	3.62	67.00	90.00	4.59
	LC (cm)	4207	54.86	5.09	29.00	78.00	9.28
	CC (cm)	4209	11.98	0.69	10.00	14.50	5.76
	BW (kg)	4464	42.62	5.36	26.00	60.00	12.58
six-month-old	BH (cm)	2517	102.20	5.37	80.00	125.00	5.25
	BL (cm)	2223	115.60	10.34	78.00	150.00	8.94
	CG (cm)	2200	131.86	11.27	100.00	165.00	8.55
	LC (cm)	1419	82.75	9.34	59.00	114.00	11.29
	CC (cm)	2172	14.85	1.02	11.00	21.00	6.87
	BW (kg)	2216	201.65	43.36	82.50	347.00	21.50

Note: BH: body height; BL: body length; CG: chest girth; LC: leg circumference; CC: cannon circumference; BW: body weight; CV: coefficient of variation.
