# Supplementary material for: Genomic Selection for Economically Important Traits in Dual-Purpose Simmental Cattle
Source: Animals (Basel). 2025 Jul 3;15(13):1960. doi: 10.3390/ani15131960 (PMC12249443; doi:10.3390/ani15131960)
Supplement: Supplementary file 1 [file animals-15-01960-s001.zip › Table S4-S6. Estimates of additive genetic variance, residual variance, and heritability for milk-production, reproduction and growth traits in dual-purpose Simmental cattle.pdf]

Table S4 Estimates of additive genetic variance, residual variance, and heritability for milk-production traits in dual-purpose Simmental cattle.

Traits	BLUP			ssGBLUP		
	σ_a^2	σ_e^2	h^2(SE)	σ_a^2	σ_e^2	h^2(SE)
305MY	858230	1915500	0.309 (0.018)	869130	1885600	0.316 (0.018)
MFP	0309.06	0.60	0.088 (0.015)	0.062	0.591	0.095 (0.015)
MFY	1292.50	3820.00	0.253 (0.019)	1310.60	3771.10	0.258 (0.018)
MPP	0.02	0.07	0.219 (0.016)	0.019	0.07	0.221 (0.016)
MPY	984.22	2233.90	0.306 (0.019)	1000.00	2199.90	0.313 (0.019)
LP	0.01	0.07	0.078 (0.015)	0.01	0.07	0.094 (0.015)
TSR	0.17	1.01	0.144 (0.018)	0.17	1.00	0.147 (0.017)
MUN	1.29	8.18	0.136 (0.016)	1.35	8.12	0.142 (0.016)
SCS	0.41	2.40	0.145 (0.016)	0.43	2.38	0.152 (0.016)

Table S5 Estimates of additive genetic variance, residual variance, and heritability for reproduction traits in dual-purpose Simmental cattle.

Traits	BLUP			ssGBLUP		
	σ_a^2	σ_e^2	h^2(SE)	σ_a^2	σ_e^2	h^2(SE)
AFCh	3116.30	7891.60	0.283 (0.032)	3422.40	7764.90	0.306 (0.032)
AFSh	3255.10	4325.50	0.429 (0.031)	3422.80	4290.00	0.443 (0.030)
AFPh	7400.10	11863.00	0.384 (0.032)	7858.60	11692.00	0.402 (0.031)
FSTCh	507.27	3307.20	0.133 (0.025)	536.51	3282.70	0.140 (0.024)
GLh	7.52	42.36	0.151 (0.031)	7.91	42.07	0.158 (0.030)
NSh	0.02	0.65	0.032 (0.017)	0.03	0.65	0.039 (0.018)
CRh	0.01	0.21	0.060 (0.030)	0.02	0.21	0.067 (0.022)
CIc	331.20	4450.80	0.069 (0.009)	324.90	4436.70	0.068 (0.009)
FSTCc	302.44	3140.40	0.088 (0.009)	295.57	3149.30	0.086 (0.009)
GLc	5.70	35.51	0.138 (0.011)	5.72	35.48	0.139 (0.011)
NSc	0.05	1.08	0.044 (0.009)	0.05	1.08	0.042 (0.009)
CRc	0.02	0.20	0.080 (0.009)	0.02	0.21	0.078 (0.009)

Table S6 Estimates of additive genetic variance, residual variance, and heritability for growth traits in dual-purpose Simmental cattle.

Stage	Traits	BLUP			ssGBLUP		
		σ_a^2	σ_e^2	σ_a^2	σ_e^2	σ_a^2	σ_e^2
newborn	BH	1.22	7.67	0.137 (0.030)	1.31	7.59	0.147 (0.031)
	BL	1.36	9.44	0.126 (0.030)	1.48	9.33	0.137 (0.031)
	CG	2.05	9.78	0.173 (0.033)	2.13	9.73	0.180 (0.033)
	LC	3.41	20.00	0.146 (0.031)	3.71	19.81	0.158 (0.031)
	CC	0.12	0.33	0.271 (0.037)	0.13	0.32	0.286 (0.037)
	BW	6.21	19.96	0.237 (0.035)	6.35	19.88	0.242 (0.035)
six-month-old	BH	6.53	16.75	0.281 (0.052)	6.80	16.60	0.291 (0.052)
	BL	22.53	47.77	0.320 (0.046)	23.08	47.66	0.326 (0.046)
	CG	22.47	51.31	0.305 (0.049)	22.31	51.85	0.301 (0.049)
	LC	29.19	40.76	0.417 (0.057)	29.85	40.47	0.425 (0.057)
	CC	0.22	0.42	0.342 (0.058)	0.20	0.44	0.318 (0.055)
	BW	515.98	683.64	0.430 (0.051)	520.56	684.68	0.432 (0.051)
