# Supplementary material for: Genomic Selection for Economically Important Traits in Dual-Purpose Simmental Cattle
Source: Animals (Basel). 2025 Jul 3;15(13):1960. doi: 10.3390/ani15131960 (PMC12249443; doi:10.3390/ani15131960)
Supplement: Supplementary file 1 [file animals-15-01960-s001.zip › Table S7. Comparison of growth traits among breeds.pdf]

Table S7. Comparison of growth traits among breeds.

	The study	Dual-purpose Simmental cattle [62, 65]	Chinese Simmental Beef cattle [58, 59]	Xianjiang brown cattle [64]	Sanhe cattle [63]	Jinnan cattle [60]	Brahman cattle [61]
BW	42.62	42.32-44.35	38.79-44.96	34.49-35.52	40.13	24.46	29.04-35.30
BH	76.72	77.56-76.83	-	66.45	76.09	67	
BL	75.08	75.73-75.81	-	75.91	74.33	60.12	74.17
CG	78.79	78.44-79.29	-	73.78	77.25	65.44	-
LC	54.86	55.78-54.15	-	-	-	-	-
CC	11.98	11.89-12.07	-	-	10.63	9.86	-
BW6	201.65	204.96-225.08	208.68	137.57-152.60	-	147.44	203.6
BH6	102.2	102.14-102.39	100.44	105.53	-	102.65	-
BL6	115.6	117.87-118.14	105.31	109.43-115.45	-	107.98	-
CG6	131.86	133.14-134.40	-	116.68-123.91	-	125.79	-
LC6	82.75	88.77-91.52	-	-	-	-	-
CC6	14.85	14.19-14.47	-	15.01	-	13.6	-
